# Dose rate correction for a silicon diode detector array

**DOI:** 10.1002/acm2.13409

**Published:** 2021-09-14

**Authors:** Andreas Jäger, Sonja Wegener, Otto A. Sauer

**Affiliations:** ^1^ Department of Radiation Oncology University of Wuerzburg Wuerzburg Germany

**Keywords:** ArcCHECK, correction, diode, dose rate, dosimetry, QA, VMAT

## Abstract

**Purpose:**

A signal dependence on dose rate was reported for the ArcCHECK array due to recombination processes within the diodes. The purpose of our work was to quantify the necessary correction and apply them to quality assurance measurements.

**Methods:**

Static 10 × 10 cm^2^ 6‐MV fields delivered by a linear accelerator were applied to the detector array while decreasing the average dose rate, that is, the pulse frequency, from 500 to 30 MU/min. An ion chamber was placed inside the ArcCHECK cavity as a reference. Furthermore, the instantaneous dose rate dependence (DRD) was studied. The position of the detector was adjusted to change the dose‐per‐pulse, varying the distance between the focus and the diode closest to the focus between 69.6 and 359.6 cm. Reference measurements were performed with an ion chamber placed inside a PMMA slab phantom at the same source‐to‐detector distances (SDDs). Exponential saturation functions were fitted to the data, with different parameters to account for two generations of ArcCHECK detectors (types 2 and 3) and both DRDs. Corrections were applied to 12 volumetric modulated arc therapy plans.

**Results:**

The sensitivity decreased by up to 2.8% with a decrease in average dose rate and by 9% with a decrease in instantaneous dose rate. Correcting the average DRD, the mean gamma pass rates (2%/2‐mm criterion) of the treatment plans were improved by 5 percentage points (PP) for diode type 3 and 0.4 PP for type 2. Correcting the instantaneous DRD, the improvement was 8.4 PP for type 3 and 0.9 PP for type 2.

**Conclusions:**

The instantaneous DRD was identified as the prevailing effect on the diode sensitivity. We developed and validated a method to correct this behavior. The number of falsely not passed treatment plans could be considerably reduced.

## 1 INTRODUCTION

In their 2004 paper, Létournau et al.^1^ write: “Pulse rate dependence of the diode should not be a concern for IMRT quality assurance (QA) because all treatments are generally delivered with the same repetition rate.” Due to the emergence of volumetric modulated arc therapy (VMAT),^2^ the situation has changed: Pulse rates *are* modulated during VMAT treatments.^3^ This means that the pulse rate dependence of radiation diodes can no longer be neglected.^4^ The dose rate dependence's (DRD) negative effect on the accuracy of QA measurements could lead to wrongful adjustments to patient treatment plans.

It has to be stressed that there are two types of dose rate: average dose rate^4^ (also called pulse rate or pulse repetition rate) and instantaneous dose rate, that is, the dose‐per‐pulse (DPP).^5^ If either of them is decreased, there can be a decrease in the sensitivity of the diode. The instantaneous DRD has been described in publications^1^, ^5^, ^6^, ^7^ dating back to the early 2000s. In the studies mentioned, the DPP was altered by changing the distance between the linear accelerator (linac) and detector. However, the photon fluence per pulse delivered from the linac itself is approximately constant, and so is the source‐to detector distance (SDD) during most QA measurements. Therefore, the instantaneous DRD and its potential negative effect on treatment plan QA have been tolerated so far, although the DPP also changes with field size and decreases dramatically in the scatter region outside the field. Furthermore, the DPP changes with depth in a phantom and as a function of distance from the machine central axis.

The physical origin of the instantaneous DRD lies within recombination processes in the diode. Incident radiation creates minority charges within a semiconductor like silicon.^8^ If these charge carriers get to the depletion zone, they will be swiped across the pn‐junction and will be collected at the opposing electrode. Part of the minority charge carriers recombine, however, reducing the overall photocurrent. Both the creation as well as recombination take place at recombination‐generation (R‐G) centers.^9^, ^10^ For a silicon diode, these are mostly impurity atoms such as platinum or crystal defects. For low injection of charge carriers, there will always be plenty of spare R‐G centers so that the recombination portion of the photocurrent remains constant. Nevertheless, the amount of R‐G centers is finite, so medium‐scale injection causes the recombination current to become dependent on instantaneous dose rate.^5^ For high injection, all R‐G centers are occupied such that the recombination portion will be independent again.

It is different for the average dose rate. A linac radiates a pulsed beam with each radiation pulse having a duration in the microsecond range. In between two pulses, there is an interruption of a few milliseconds, so a linac's typical pulse frequency is around 100 to 1000 Hz. In order to modulate the average dose rate, the pulse frequency is varied, while the pulses stay the same in terms of shape and width (i.e., the DPP stays constant). Therefore, the average dose rate is frequently varied during VMAT, also causing a change of diode sensitivity.

The average DRD is also driven by minority charge carriers being caught and let go. Although the exact mechanism is not fully understood,^11^ the current description states that the charge‐capture processes are dominated by traps,^4^ which are different from R‐G centers. While R‐G centers result from impurity atoms, traps originate from imperfections in the crystal lattice^12^ such as dangling bonds.^13^ R‐G centers catch and release charges on the same time scale. Traps, on the other side, capture electrons and holes fast (milliseconds), but they slowly release the charges (seconds to minutes). If the repetition rate is fast, the trap leakage is low. Most traps are filled with charges that are created by the first radiation pulses. So for subsequent pulses, they remain closed. Hence, there is a little reduction of the overall photocurrent. For slow repetition rates, however, there is enough time for some traps to reopen for those charges that have been generated by secondary, tertiary (etc.) radiation pulses. Hence, modulating the pulse rate causes a variation of photocurrent.

While both the instantaneous as well as the average DRD have been described by several researchers (e.g., Rosenfeld et al.),^11^ so far, correction mechanisms for silicon diode detectors were scarce. It was suggested to control the instantaneous DRD by tailoring the R‐G‐centers such that the electrical resistivity of the silicon substrate is decreased or that the mean lifetime of the minority carriers is reduced.^4^, ^5^, ^14^ In terms of the average DRD, a built‐in correction has been released with a software update of the SRS MapCHECK (Sun Nuclear) detector, which was validated by Ahmed et al.^15^ In another study, Kozelka et al.^16^ stated that the ArcCHECK (Sun Nuclear) diode array can be calibrated with the expected mean pulse rate as a correction factor because the pulse rate dependence they observed was below 1%.

In our clinic, we use two ArcCHECK devices for QA of VMAT plans too. We experienced a large drop in our pass rates when switching plan QA from an ArcCHECK with type‐2 diodes to one equipped with type‐3 detectors. Those coincided with larger DRDs observed for the type 3 detector. Therefore, we quantified the DRDs of the two ArcCHECK detector arrays. Based on these results, we developed a correction code with individual correction factors for each device and type of DRD (average and instantaneous). Finally, the impact of the correction on treatment plan measurements was evaluated by comparing uncorrected and corrected measurement data with the dose distribution calculated by the treatment planning system (TPS).

In this work, we did a comparative study of average and instantaneous DRD for ArcCHECK diodes. We wrote a correction code that can be easily run on measured QA data. The influence of the correction on QA results was investigated.

## 2 MATERIALS AND METHODS

The DRDs of two different ArcCHECK detectors were investigated. An older one (SN 6622416) equipped with “diode type 2” and the latest version (SN 6317512) equipped with “diode type 3.” Both types are SunPoint™ (Sun Nuclear) diodes, the semiconductor material being silicon. According to the manufacturer (personal communication, 02.06.2021), the two types of diodes are identical, but they differ in terms of soldering. As a consequence, the latest version “type 3” has a higher sensitivity and therefore detects more counts per unit of time. Due to their different behavior, and in order to discriminate between the two detectors we studied, we keep referring to the diodes as “type 2” and “type 3.” For all measurements, the cavity plug was inserted.

### 2.1 Average dose rate

Static 6‐MV photon beams, provided by an Elekta Synergy linear accelarator (Elekta) were used for irradiating the ArcCHECK detector. The field size was 10 × 10 cm^2^ at the isocenter. The SSD distance was SDD=(100 − 13.3 + 2.9) cm =89.6 cm, which results from the difference of the source‐to‐isocenter distance (100 cm), the ArcCHECK radius (13.3 cm), and the diode depth (2.9 cm).^17^ Either 100 or 200 MU were delivered. The average dose rate (i.e., pulse‐repetition rate) was varied, starting from the maximum available dose rate of approximately 500 MU/min and then by reducing it by halving the pulse repetition rate for each subsequent measurement, until the lowest rate was approximately 30 MU/min. In order to gain a reference signal, a 0.125 cm^3^ ion chamber Semiflex 31010 connected to a Multidos Electrometer (both PTW‐Freiburg) was placed inside the central cavity of the ArcCHECK phantom.

Since all ArcCHECK diodes are read out every 50 ms, all following sensitivity dependencies are stated as a function of counts/50 ms. The integral of counts/50 ms over time yields the cumulative count.

### 2.2 Instantaneous dose rate

**FIGURE 1:**
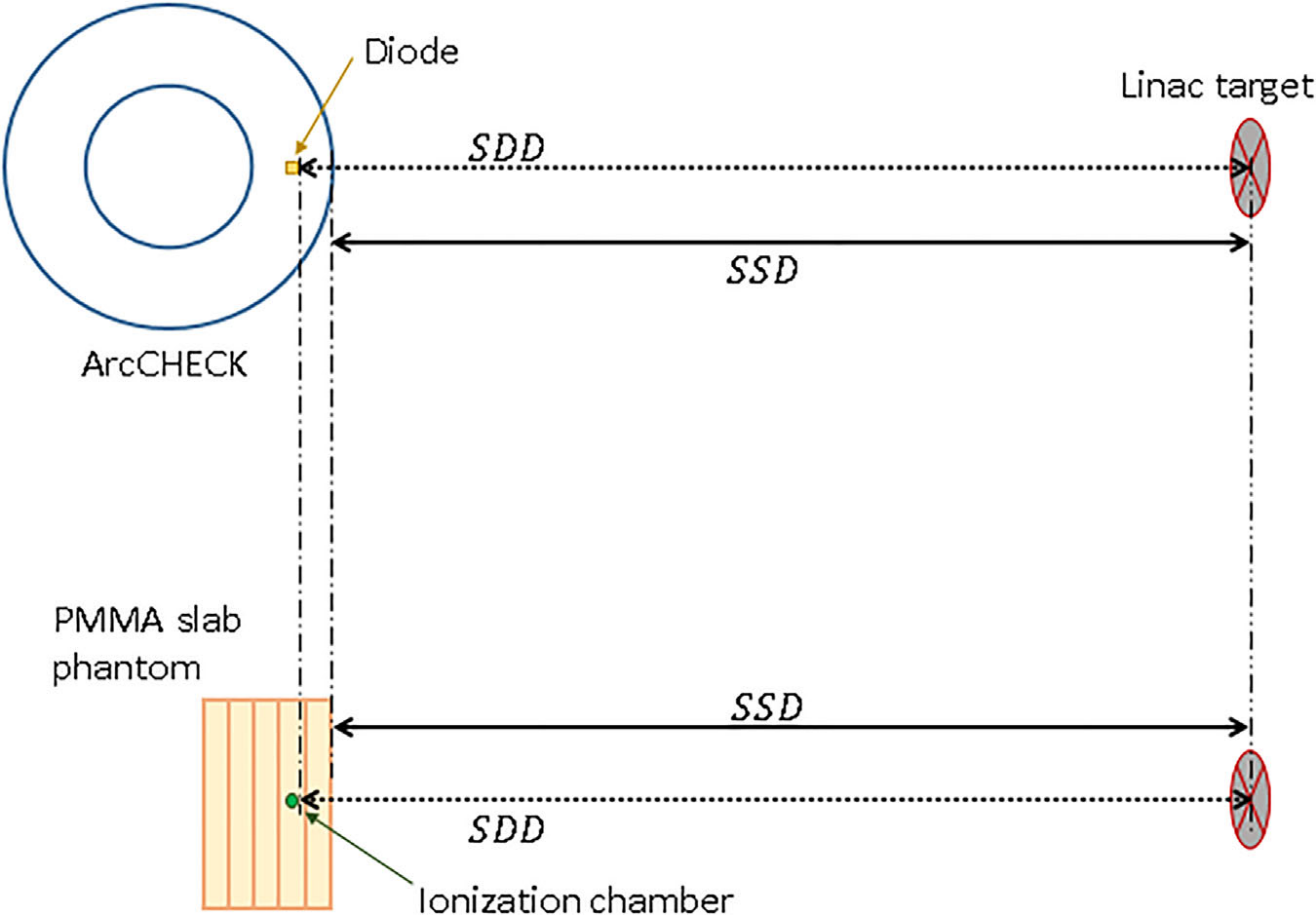
Qualitative drawing of the geometric setup used for measuring the instantaneous dose rate dependence

Additionally, the instantaneous DRD was studied by varying the SDD between 69.6 and 359.6 cm while keeping the pulse repetition frequency constant at the maximum 500 MU/min. The jaw setting was kept a 10 × 10 cm^2^ field. In order to get the reference dose at each measurement point, we repeated the series, only now we replaced the ArcCHECK with a 10‐cm thick PMMA slab phantom. The ion chamber (Semiflex 31010) was inserted into a cavity at a depth of 2.9 cm, that is, the depth of the diode array below the surface of the ArcCHECK phantom (see Figure 1). By using a laser distance meter (Leica Geosystems), theSSDwas monitored.

### 2.3 Data evaluation and curve fitting

The mean total count of 12 central diodes within the 10 × 10 cm^2^ field was evaluated for each measurement of average and instantaneous dose rate using Sun Nuclear Corporation (SNC) Patient version 6.7.3 (Sun Nuclear). Each mean value was normalized to the corresponding ion chamber signal. Counts/50 ms were calculated by averaging the plateau count/50 ms over the effective measuring time and the 12 central diodes.

Due to the fact that with increasing dose rate the response converges to a maximum, an exponential saturation function was chosen to fit the relative diode response y:

(1) $$y\;=\;c-a\cdot e^{-\mathrm{bx}},$$

where x is the dose rate expressed in counts/50 ms, and a, b, and c are fitting parameters. When x=0 it follows that y=c−a, and thus the parameter a has an impact on the vertical intercept of the function, whilec is the saturation value (y=c for x→∞). The parameter b is a measure for the curvature. The data were fitted using the “lsqcurvefit” (i.e., least‐square curve fitting) function from MATLAB.^18^

In the following, the correction obtained from the central 12 diodes was applied to each diode assuming a general validity.

### 2.4 Correction code

When performing a measurement with the ArcCHECK detector, usually three types of files are saved: first, an “ArcCHECK movie” file (.acm‐file) with time‐dependent information; second, an “ArcCHECK movie light” file (.acml‐file) with reduced information; and third, a text file (.txt‐file) with all final values.

The .acml‐file is needed for specific software functionalities but is not used for our proposed correction. The .txt‐files contain the cumulative values of raw counts, software‐specific corrected counts, and dose per diode. The software‐specific corrections are derived from the .acml‐file to account for the diode angular dependence and field size for each time interval. The .txt‐file is loaded into the SNC Patient software for analysis.

While the .txt‐files account for the spatial resolution, they only provide cumulative values of dose and counts. However, in order to correct the DRD of every count/50 ms of every diode, the “ArcCHECK movie” files (.acm‐files) need to be used since they contain time‐dependent information.

The principal mechanism of the correction code is as follows: $x_{n,\;t}$ is the differential raw count detected by the n‐th diode at a given update t (the elapsed time between two subsequent updates is always 50 ms). Taking the DRD into account, the raw count is corrected to $x_{n,t,dose\;rate\;corrected}$ by

(2) $$\;x_{n,t,\mathrm{dose}\;\mathrm{rate}\;\mathrm{corr}\mathrm{ected}}=\frac{x_{n,\;t}-x_{n,\;\mathrm{Back}\mathrm{grou}\mathrm{nd}}}{y\left(x_{n,\;t}\right)}\;\cdot cf_{n},$$

where $y(x_{n,\;t})$ is given by Equation (1) and depends on the type of dose rate change on the detector. Furthermore, $x_{n,\;Background}$ is the dark current (background) detected by diode n, and$\;cf_{n}$ is the array calibration factor. For $y\;(x_{n,\;t})=\;1$, Equation (2) is equivalent to the expression for the “net relative dose” stated per default in the .acm‐files. Accordingly, $y(x_{n,\;t})\neq 1$ accounts for any correction of the DRD.

Summing up, over time t yields the corrected cumulative raw count $RC_{n,dose\;rate\;corrected}$ for diode n:

(3) $$\;\sum_{t\;=\;0}^{t_{\mathrm{end}}}x_{n,t,\mathrm{dose}\;\mathrm{rate}\;\mathrm{corr}\mathrm{ected}}=RC_{n,\mathrm{dose}\;\mathrm{rate}\;\mathrm{corr}\mathrm{ected}.}$$

Finally, the intrinsic geometric corrections (angular and filed size) that ArcCHECK does by itself have to be considered by a diode‐specific factor $\varphi_{n}:$

(4) $$\;CC_{n,\mathrm{dose}\;\mathrm{rate}\;\mathrm{corr}\mathrm{ected}}=RC_{n,\mathrm{dose}\;\mathrm{rate}\;\mathrm{corr}\mathrm{ected}}\;\cdot \varphi_{n}.$$

Here, $CC_{n,dose\;rate\;corrected}$ are the cumulative corrected counts (both geometric corrected as well as dose rate‐corrected). The conversion factor can be obtained from the .txt‐files as $\varphi_{n}=\frac{CC_{n,old}}{RC_{n,old}}\;$, where $RC_{n,old}$ are the uncorrected cumulative raw counts, and $CC_{n,old}\;$are the former corrected counts, which only feature the geometric corrections. Replacing $CC_{n,old}$ with $CC_{n,dose\;rate\;corrected}$ in the .txt‐files allows for the comparison of corrected QA with the simulated plans.

Note that the different diode positions with respect to beam axis (angular dependence) and field size are corrected intrinsically by the ArcCHECK software. The intrinsic correction factors (see diode‐specific factor $\varphi_{n}$) are unchanged by our dose rate correction.

### 2.5 Quantitative analysis of corrected QA plans

Using the four different versions of the exponential saturation function y for both diode types and DRDs, measurements of treatment plans per detector were corrected. We picked 12 plans per detector from our archives that had been planned with Pinnacle^3^ Version 16.2.1 (Philips Medical Systems) and been measured during routine QA, which had scored below average in terms of gamma pass rates.^19^, ^20^ The average dose rate during the measurements typically varied between ∼100 and 500 MU/min. The dose at the entry and exit regions of the detector differed by a factor of ∼4, which mainly accounts for the instantaneous DRD. In other words, the 12 planes we chose were not special with regard to dose rate. We rather chose them because their QA measurements had low gamma pass rates. In the routine QA, both detectors were calibrated by 10 × 10 cm^2^ fields at maximal pulse rate, all plans were delivered by the linac with an energy of 6 MV.

Global gamma pass rates were evaluated with SNC Patient version 6.7.3 (Sun Nuclear) at a low‐dose threshold of 10% in absolute dose mode. The count of a diode is classified as “passed,” if its value lies within the chosen criterion, either 2%/2‐mm tolerance, compared to the TPS‐calculated reference signal, or 3%/2 mm or 3%/3 mm. Especially the 2%/2 mm as well as the 3%/2 mm criteria are a typical benchmark because they resemble tolerances of dose optimization algorithms.^7^ The corrected evaluations were compared to the native ones, and the change in gamma pass rates (percentage points (PP)) was analyzed.

## 3 RESULTS

**FIGURE 2:**
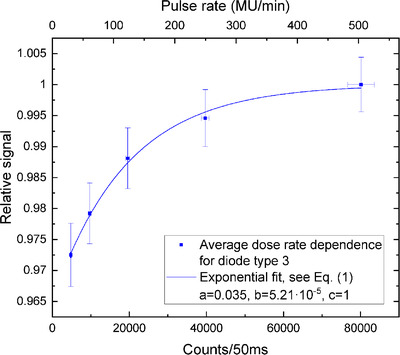
Diode type 3 response versus average dose rate. The goodness of fit was evaluated to be *R* ^2 ^= 0.9962

**FIGURE 3:**
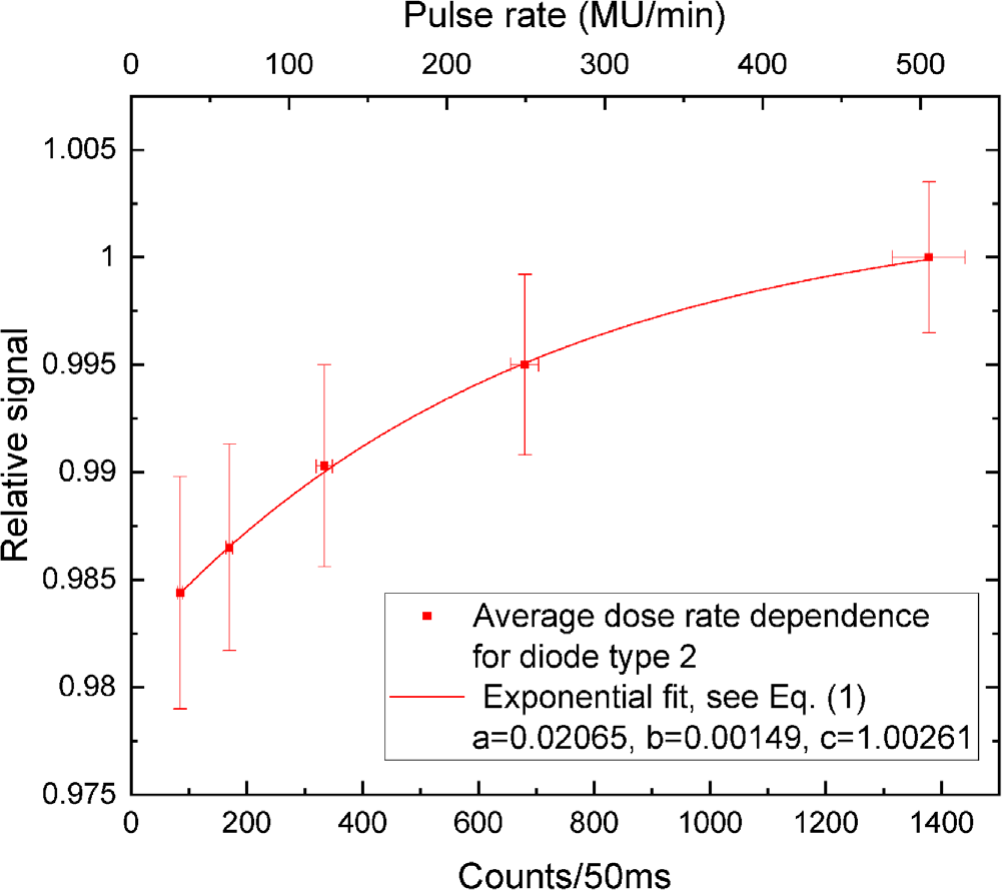
Diode type 2 response versus average dose rate (fit: *R* ^2 ^= 0.9995)

**FIGURE 4:**
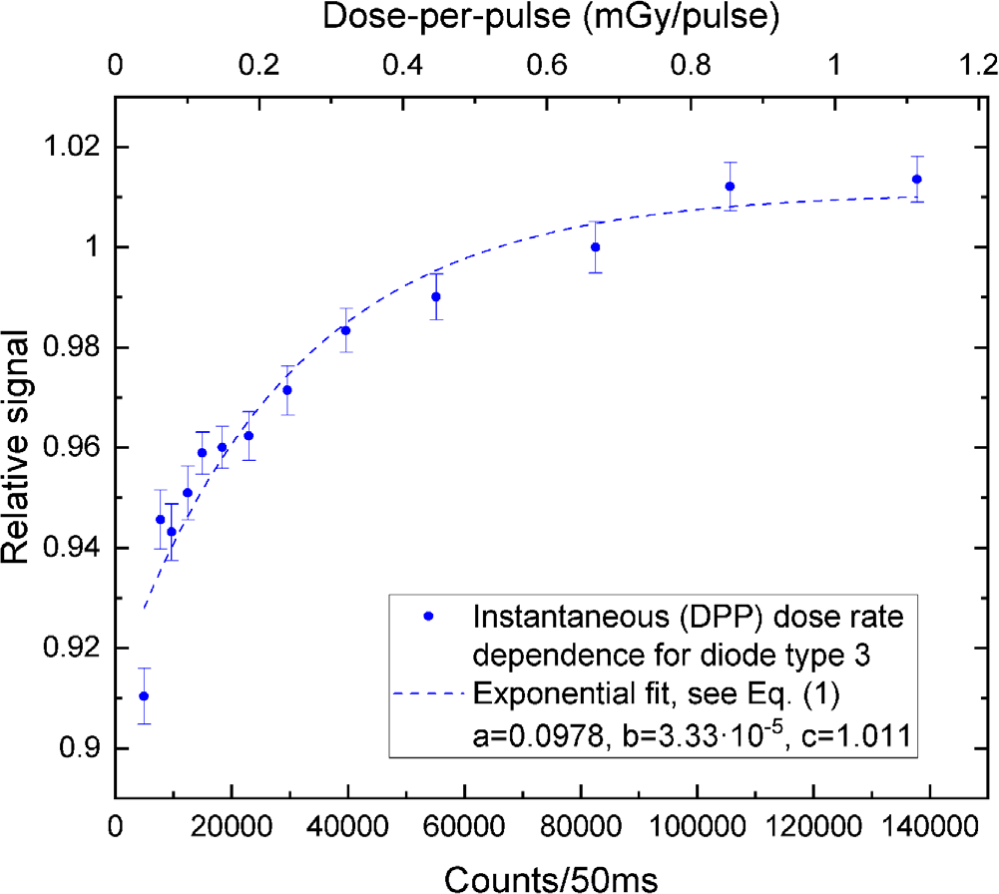
Diode type 3 response versus instantaneous dose rate (fit: *R* ^2^ = 0.9436)

**FIGURE 5:**
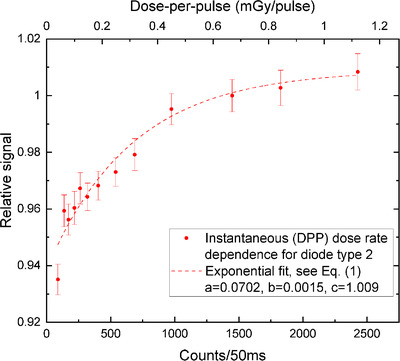
Diode type 2 response versus instantaneous dose rate (fit: *R* ^2^ = 0.9445)

The diode sensitivity decreased with the sinking dose rate (Figures 2, 3, 4, 5). The uncertainty bars of the relative signal indicate one standard deviation for averaging over 12 diodes. The parameters resulting from the fit of the exponential, see Equation (1), are given in Table 1 for the four different set‐ups.

**TABLE 1:** Fit parameters a, b, and c for both dose rate dependencies and diode types

Correction model	Diode type	γ (2%/2 mm)	γ (3%/2 mm)	γ (3%/3 mm)
Average d. r.	3	(+5.0±3.1) PP	(+2.9±2.3) PP	(+1.7±1.5) PP
Average d. r.	2	(+0.4±0.4) PP	(+0.2±0.3) PP	(+0.1±0.3) PP
Instantaneous d. r.	3	(+8.4±6.2) PP	(+4.9±4.2)PP	(+2.5±2.6) PP
Instantaneous d. r.	2	(+0.9±1.1)PP	(+0.5±0.8) PP	(+0.2±0.6)PP

Abbreviations: PP, percentage points; d.r., dose rate.

### 3.1 Average DRD

**FIGURE A1:**
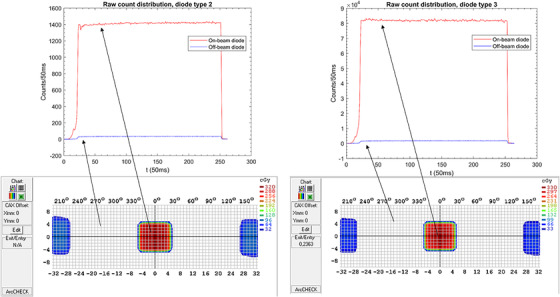
Comparison of raw counts for types 2 and 3 diodes (the one is on‐beam, the other is off‐beam) for irradiation with a 10 × 10 cm^2^ field of 6‐MV energy, with 200 MU and 500 MU/min repetition rate. Overview dose distributions were retrieved from SNC Patient

For the average DRDs shown in Figures 2 and 3, the sensitivity decreases by 2.8% for diode type 3 and by more than 1.5% for diode type 2. We repeated the pulse rate (MU/min) measurements three times per detector. Averaging over the sessions, one standard deviation yielded the dose rate uncertainties. While the maximal deviation from the reference signal differs by a factor of 2 between both diode types, there is also a substantial difference in counts/50 ms. The counts per dose are much smaller for diode type 2 because they are soldered differently to the electric contacts than in “type 3” ArcCHECK detectors. Therefore, type‐2 diodes generally have a lower sensitivity, which is illustrated in the comparison of raw counts for an on‐beam and an off‐beam diode of types 2 and 3 (see the Figure A1). The on‐beam plateau raw count detected by a type‐2 diode is 1400, while for the type‐3 diode it is 80 000 (sensitivity of type‐2 diodes is lower by a factor of ∼57).

### 3.2 Instantaneous DRD

Converting dose rate into counts/50 ms allows for the comparison of average versus instantaneous DRD (see Figures 4 and 5). In accordance with the average DRD, the sensitivity decrease is larger for diode type 3, compared to diode type 2. However, the instantaneous DRD is much larger overall: the diode response drops by 9% for diode type 3 and by 6.5% for type 2. The relative signal was normalized to 1 for SDD= 89.6 cm (source‐to‐phantom‐center distance of 100 cm).

### 3.3 Quantitative analysis of corrected QA plans

Running the correction code with individual parameters a, b, and c on 12 QA measurements of treatment plans per detector, the gamma pass rates could be improved (see Table 2) for each correction model:

**TABLE 2:** Mean relative improvement (PP) of the gamma pass rates for the corrected quality assurance data, compared to the native measurements. A total of 12 treatment plans were tested, the same ones for each ArcCHECK device (diode type)

Correction model	Diode type	*a*	*b*	*c*
Average dose rate	3	0.0350 ± 0.0017	(0.0521 ± 0.0068)∙10−3	1.0000 ± 0.0011
Average dose rate	2	0.02065 ± 0.00037	(1.490 ± 0.094)∙10−3	1.00261 ± 0.00047
Instantaneous d. r.	3	0.0978 ± 0.0075	(0.0333 ± 0.0068)∙10−3	1.0110 ± 0.0056
Instantaneous d. r.	2	0.0702 ± 0.0058	(1.50 ± 0.39)∙10−3	1.0090 ± 0.0062

An example shall illustrate the improvement of gamma pass rates due to the correction: The quota of diodes (e.g., type 3) that have “passed” the 3%/2 mm criterion is 90.2% for a given native (uncorrected) plan. After correcting the instantaneous DRD, 95.3% of the diodes “pass,” which accounts for an improvement of +5.1 PP.

Although the improvements are bigger for the instantaneous dose rate correction, compared to the average dose rate, they come with wider variance. The standard deviation of the relative improvement of 12 QA plans corrected was 6.2 PP for instantaneous dose rate (2%/2 mm criterion, diode type 3), whereas the standard deviation for average dose rate was only half of this: 3.1 PP. For the instantaneous dose rate, 3 out of 12 QA plans actually worsened by ≤1 PP after the correction with respect to the 3%/3 mm criterion, whereas just 1 out of the identical 12 QA plans did so for the average dose rate. For type‐2 diodes, the same 2 out of 12 QA plans worsened after the correction of the average as well as the instantaneous DRD.

## 4 DISCUSSION

Two types of DRDs affect measurements of diodes built in the ArcCHECK detector. Whereas the manufacturer states the DRD to be ±1% over the range 150 to 1400 MU/min^17^ as well as for 600‐fold changes in DPP,^21^, ^22^ we demonstrated that—especially for type‐3 diodes—the instantaneous DRD is bigger than that. It can be deduced that although the diodes of types 2 and 3 themselves are equal, the different ways of them being soldered within the ArcCHECK devices not only makes type‐3 diodes generally more sensitive (by a factor of ∼57, compared to type 2) but also leads to a different behavior regarding dose rate. We confirmed that the average DRD varies by ±1% within the given range^17^ between 150 and 1400 MU/min but exceeds it for pulse rates smaller than 150 MU/min. Small pulse rates should not be excluded from commissioning because pulse rates can actually drop below 150 MU/min during VMAT treatments.

The average DRD was varied by reducing the pulse rate of the linac, starting from 500 MU/min. Regardless of pulse rate, the diode signal per unit time (counts/50 ms) integrated over time should result in the same signal. In contrast to that, we found that the diode response decreased with a decrease in MU/min by up to 2.8% for diode type 3 and by up to 1.5% for diode type 2. Our findings agree with earlier measurements of the average DRD: Houweling et al.^23^ observed a maximal diode response difference of 2.1%. By normalizing the diodes to the median reading at 128 MU/min, they then quantified the tolerance to be ±1% and in that way confirming the pulse rate dependence stated by the manufacturer. They measured with an ArcCHECK for use in a magnetic resonance (MR) linac but did not specify the generation (diode type).

Letourneau et al.^1^ measured a 2% sensitivity variation due to the average DRD. Jursinic^4^ quantified it to be 2%–5%. Both studies were performed on the MapCHECK detector, being equipped with SunPoint™ diodes (both Sun Nuclear), the same type of diode built into the ArcCHECK detector. It can be assumed that the soldering of the diodes within MapCHECK is again different from the types 2 and 3 ArcCHECK detectors, yet their results further indicate that the average DRD can generally exceed ±1%.

The instantaneous dose rate (DPP) dependence was measured by changing the SDD and thus by varying the DPP. We found that the diode sensitivity decreases with decreasing DPP. The physical origin of this effect has been described by Shi et al.,^5^ who observed that the recombination of minority carriers is dependent on instantaneous dose rate on a medium scale of injection, with the recombination current reducing the photocurrent. The resulting deviations from the nominal signal (SDD= 89.6 cm and 500 MU/min) are larger for the instantaneous dose rate, compared to the average dose rate, indicating that this is the main effect: The sensitivity drops by up to 9% for diode type 3 and 6.5% for diode type 2. Measurements closer to the focus revealed that the DRD indeed saturated as theoretically predicated by our exponential fit model (see Equation 1). Earlier studies^1^, ^7^ of SunPoint™ diodes support our results that the instantaneous DRD is larger than the average dose rate. However, results on the DPP dependence have not been uniform. Ahmed et al.,^24^ who studied a type 2 ArcCHECK, estimated the change in response to be around 2%. Chaswal et al.,^7^ who presumably had the predecessor model (original version 1), measured a maximal difference in response of 7.1%. Note that both of them used similar SDDs, but only went as far away from the source as 120 cm, which is about the maximal distance that can be achieved with the array on a typical couch. Additionally, diodes from other manufacturers also exhibited a decrease of sensitivity with decreasing instantaneous dose rate.^5^, ^6^

We proposed an exponential saturation function to fit the DRD. MATLAB code was written to correct QA data after their measurement, with individual fit parameters depending on the diode type and type of DRD. Regarding the 2%/2 mm criterion, after correcting the DPP dependence, the mean gamma pass rates improved by +8.4 PP for diode type‐ 3 and +0.9 PP for diode type 2. After correcting the average DRD, mean gamma rates improved by +5.0 PP for diode type 3 and by +0.4 PP for diode type 2. For the 3%/2 mm criterion, an improvement of +4.9 PP could be achieved when correcting the instantaneous DRD of diode type 3 and +0.5 PP for diode type 2.

The difference in improvement between both diode types is quite striking since type‐3 diodes have a larger DRD than type‐2 diodes. This is the reason why the gamma pass rates decreased, often to an unacceptable value, when switching from a type‐2 to a type‐3 device. Therefore, the older version (type 2) of the ArcCHECK detector is better than the latest version (type 3) with regard to dose rate stability. In clinical routine, the dose rate correction should be applied when both the gamma 2%/2 mm and the 3%/2 mm criterion are failed (passing rate below 95% and 97%, respectively). In our experience, it is acceptable to use the type‐2 version without having to apply the dose rate correction. In contrast to that, when using a type‐3 version of the ArcCHECK, the DRD needs to be corrected.

## 5 CONCLUSION

The instantaneous DRD turned out to be the prevailing reason for a decrease in diode sensitivity with decreasing dose rate. We developed a correction code to make up for the resulting inaccuracy that can be used on ArcCHECK raw data. A 12 QA measurements of treatment plans per detector were corrected, with individual parameters depending on the diode type and type of DRD. The improvements of gamma pass rates after correcting the instantaneous DRD are larger, compared to the improvements when correcting the average DRD.

We intend to use our code to correct the instantaneous DRD of QA measurements of treatment plans. Future research and software development could aim at identifying the type of DRD of individual data points from QA measurements and then by combining both correction models. Presumably, the pulse rate dependence is the prevailing effect in the primary incident region, whereas the instantaneous DRD dominates for scattered photons, for example, in the exit region.

## AUTHOR CONTRIBUTION

Sonja Wegener came forward with the conception of the study, collected and interpreted the data. Andreas Jäger collected the data, analyzed the measurements, and coded the correction scripts. Otto A. Sauer interpreted the data. Andreas Jäger wrote the article draft, which was critically reviewed by Sonja Wegener and Otto A. Sauer and finally approved for submission.

## Data Availability

The data that support the findings of this study are available on request from the corresponding author. The data are not publicly available due to privacy restrictions.
